# The ribosomal protein P0A is required for embryo development in rice

**DOI:** 10.1186/s12870-023-04445-y

**Published:** 2023-10-05

**Authors:** Zhenyi Chang, Xia Wang, Xiaoying Pan, Wei Yan, Wenshi Wu, Yi zhuang, Zhiai Li, Dan Wang, Shuting Yuan, Chunjue Xu, Zhufeng Chen, Dongfeng Liu, Zi Sheng Chen, Xiaoyan Tang, Jianxin Wu

**Affiliations:** 1 Guangdong Provincial Key Laboratory of Biotechnology for Plant Development, School of Life Sciences, South China Normal University, Guangzhou, 510631 China; 2 School of Pharmaceutical Sciences, Guangzhou University of Chinese Medicine, Guangzhou, 510006 China; 3 Shenzhen Institute of Molecular Crop Design, Shenzhen, 518107 China; 4 Shenzhen Agricultural Technology Promotion Center, Shenzhen, 518055 China

**Keywords:** Ribosomal P0 protein, Embryo lethality, P stalk, Gene duplication, Oryza sativa

## Abstract

**Background:**

The P-stalk is a conserved and vital structural element of ribosome. The eukaryotic P-stalk exists as a P0-(P1-P2)_2_ pentameric complex, in which P0 function as a base structure for incorporating the stalk onto 60S pre-ribosome. Prior studies have suggested that *P0* genes are indispensable for survival in yeast and animals. However, the functions of *P0* genes in plants remain elusive.

**Results:**

In the present study, we show that rice has three *P0* genes predicted to encode highly conserved proteins OsP0A, OsP0B and OsP0C. All of these P0 proteins were localized both in cytoplasm and nucleus, and all interacted with OsP1. Intriguingly, the transcripts of *OsP0A* presented more than 90% of the total *P0* transcripts. Moreover, knockout of *OsP0A* led to embryo lethality, while single or double knockout of *OsP0B* and *OsP0C* did not show any visible defects in rice. The genomic DNA of *OsP0A* could well complement the lethal phenotypes of *osp0a* mutant. Finally, sequence and syntenic analyses revealed that *OsP0C* evolved from *OsP0A*, and that duplication of genomic fragment harboring *OsP0C* further gave birth to *OsP0B*, and both of these duplication events might happen prior to the differentiation of *indica* and *japonica* subspecies in rice ancestor.

**Conclusion:**

These data suggested that *OsP0A* functions as the predominant *P0* gene, playing an essential role in embryo development in rice. Our findings highlighted the importance of *P0* genes in plant development.

**Supplementary Information:**

The online version contains supplementary material available at 10.1186/s12870-023-04445-y.

## Background

Protein translation is a fundamental biological process, which is carried out by ribosome. A typical ribosome consists of two subunits, a large subunit and a small subunit, both of which are comprised of ribosomal RNAs and proteins in all organism [1, 2]. The large subunit has a conserved distinctive morphological feature, the stalk protuberance, that is responsible for recruiting translation factors and stimulating translation [3]. In eukaryotes, the stalk exists as a P0-(P1-P2)_2_ pentameric protein complex [4]. P0, P1 and P2 are all acidic ribosomal proteins, and all of them were identified as phosphorylated proteins in vivo, and for this reason, they were designated as P protein [5]. The P0 protein forms the base of the stalk. It contains two conserved domains including the RNA-interacting domain at the N-terminus and the P1/P2 interacting domain (P-domain) at the C-terminus [6–9]. The RNA-interacting domain is functionally conserved. It directly interacts with 28S rRNA and is responsible for anchoring the P0-(P1-P2)_2_ complex into the 60S subunit [8]. The ribosomal proteins from P1/P2 families form P1-P2 hetero-dimers, in which the N-terminus of P1/P2 proteins are responsible for the dimerization and interaction with the P-domain of P0 protein [9]. Yeast has only one *P0* gene and four *P1/P2* genes *P1A*, *P1B*, *P2A* and *P2B*. The P1A–P2B protein complex was proposed to be the key element in stalk formation, whereas the P1B–P2A protein complex was implicated in regulation of stalk function [7]. Loss of function of P0 gene resulted in a deficiency in active 60 S subunits, thus making the yeast cell nonviable [6]. The P1/P2 disrupted yeast strains were viable but grew with a doubling time threefold higher than wild-type cells [10]. However, ribosomes depleted of P1/P2 proteins were impaired in translation of some specific mRNAs, and translation fidelity at elongation and termination steps [10, 11]. These results suggested that P0 was essential for cell survival, while P1 and P2 were dispensable for cell viability [12]. However, a well-organized P0-(P1-P2)_2_ complex was required for optimal ribosomal activity and cell survival [12].

P0 family proteins are functionally conserved in eukaryotes. The yeast mutant lacking *P0* gene could be complemented by the *P0* genes from animal [13–15]. The first plant *P0* gene was cloned as a light-induced gene from the callus of *Chenopodium rubrum*, and was proposed having a similar function as that of *P0* family genes [16]. A comprehensive research on the maize 60S ribosomal stalk identified a P0 protein and four distinctive forms of P1/P2 family proteins, including P1, P2a, P2b and a plant specific P1/P2-like protein named as P3 [17]. The phosphorylation levels of P0 and P1/P2 proteins were upregulated by oxygen deprivation, therefore affecting ribosome assemble, indicating P proteins might be involved in stress response [17]. A cDNA of rice *P0* gene was characterized, and deduced to encode a 34-KD protein [18]. Although plant *P0* genes were cloned and reported more than two decades ago [16–18], the biological functions of plant *P0* genes remain elusive.

Here, we characterized the three *P0* (*OsP0A*, *OsP0B* and *OsP0C*) genes in rice, and revealed that *OsP0A* is essential for rice embryo development, while *OsP0B* and *OsP0C* were dispensable in plant development. Moreover, syntenic and sequences analyses revealed that the rice ancient *P0* gene underwent two duplication events and *OsP0A* was proposed to be the ancestral *P0* gene. In all, our results highlighted the critical function of Os*P0A* in rice development.

## Results

### Phylogenetic analysis of plant ribosomal protein P0s

**Fig. 1:**
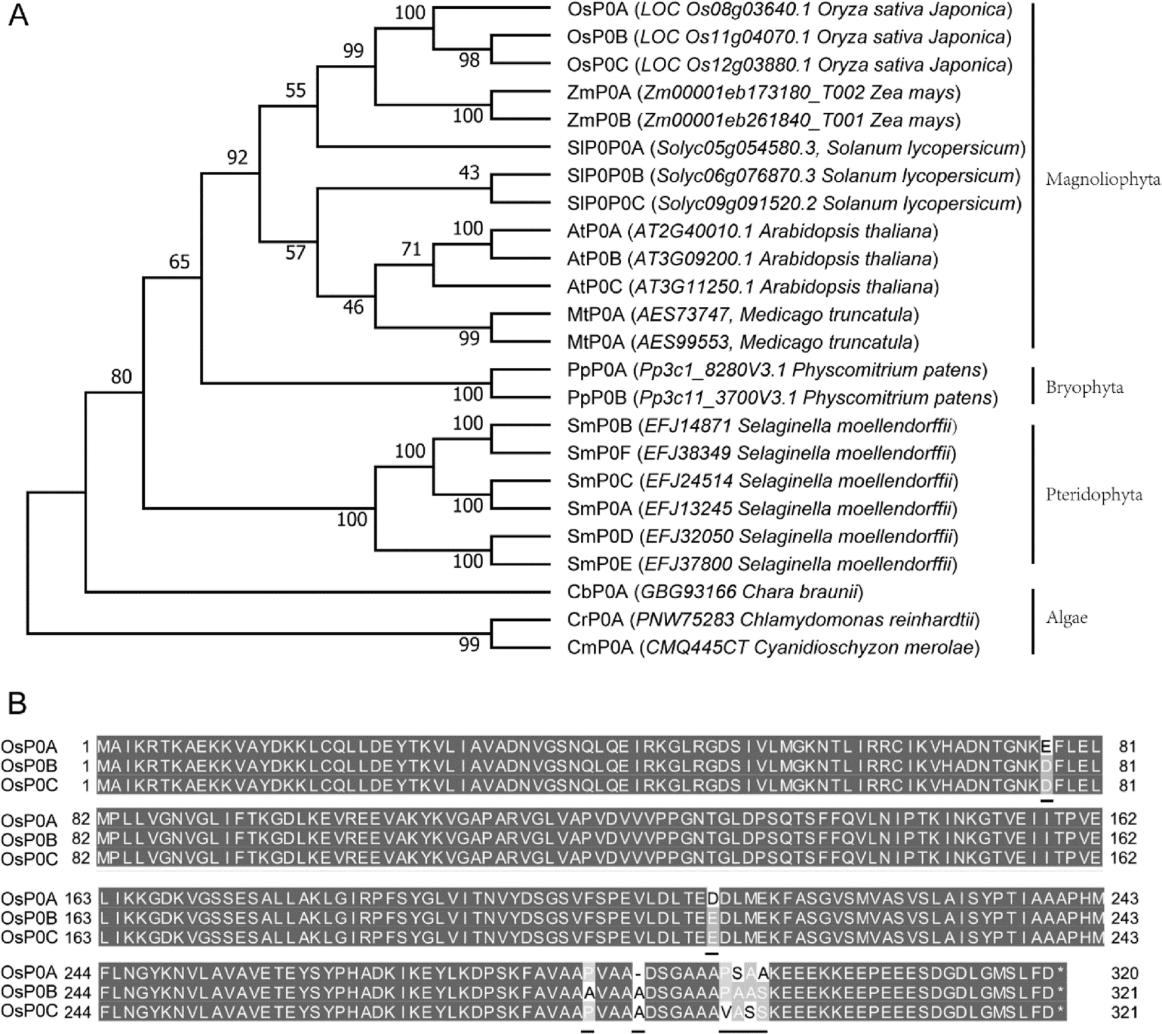
Phylogenetic analysis of plant P0 proteins. P0 proteins were identified from various plant species, and the phylogenetic tree was built by MEGAX (**A**). The rice P0 protein sequences were aligned by Clustal X and the differences were highlighted with dark underline (**B**)

Homolog search of yeast P0 protein against the Nipponbare rice genome, identified three distinctive P0 proteins designated as P0A, P0B and P0C, which were encoded by *LOC_Os08g03640*, *LOC_Os11g04070*, and *LOC_Os12g03880*, respectively. To investigate the evolutionary patterns of plant P0 proteins, other 21 P0 proteins were identified from the nine representative species including unicell algae *Cyanidioschyzon merolae* and *Chlamydomonas reinhardtii*, charophyte *Chara braunii*, pteridophyte *Selaginella moellendorffii*, bryophyte *Physcomitrium patens*, and magnoliophyte *Solanum lycopersicum*, *Arabidopsis thaliana*, *Medicago truncatula* and *Zea mays*. Multiple sequences alignment and phylogenetic analysis showed that P0 proteins are highly conserved in plant kingdom (Fig. S1, Fig. 1A). Interestingly, each of the representative algae species has only one P0 gene, whereas each of the land plant species has at least two P0 genes (Fig. 1A), indicating that the plant *P0* genes might be duplicated during the transition of plants from aquatic to terrestrial environments. The paralogues of rice P0 proteins are very similar in protein sequence. OsP0A shares 96% and 95% protein sequence identities with OsP0B and OsP0C respectively, while OsP0B and OsP0C exhibited 99% identity (Fig. 1B), indicating that *OsP0B* and *OsP0C* are more closely related during gene evolution.

### Subcellular localization of OsP0s proteins

**Fig. 2:**
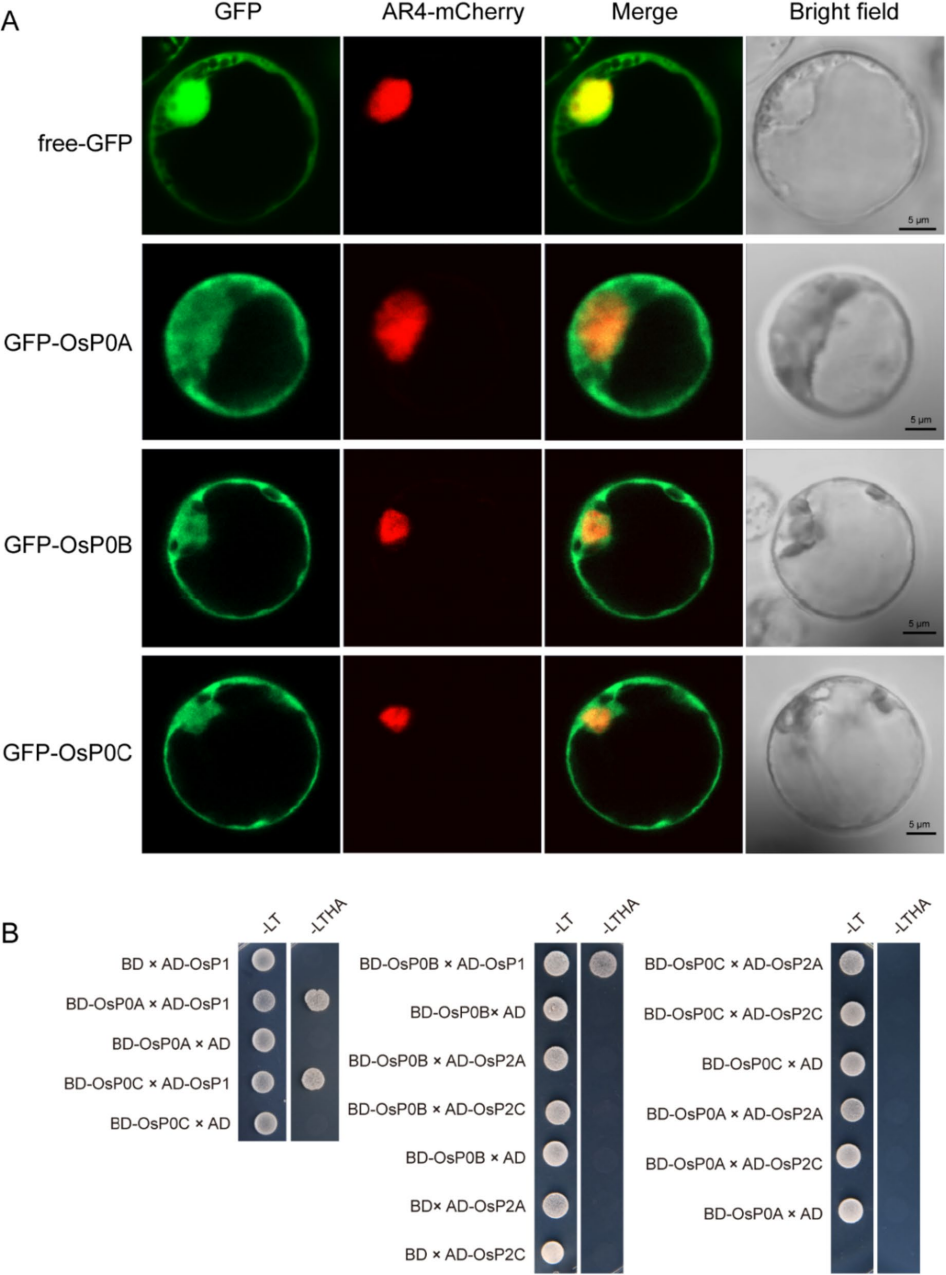
Subcellular localization of OsP0s and the protein–protein interactions between OsP0s and OsP1/P2 proteins. (**A**) The localization of GFP-OsP0A, GFP-OsP0B and GFP-OsP0C in rice protoplast. Free GFP was used as a control. The Ar4-mCherry was used as the marker of nucleus. Scale bars = 5 μm. (**B**) Interaction analyses of P0 proteins with their partner proteins by the yeast two hybrid assay. -LT represents the synthetic medium lacking Leu and Trp, and -LTHA represents the synthetic medium lacking Leu, Trp, His and Ade

Proteins localized in appropriate cell compartments is vital for exerting their functions. Previous studies revealed that the yeast ScP0 protein was localized in the cytoplasm [19], and the C-terminus of ScP0 was critical for the protein localization [19]. OsP0A, OsP0B and OsP0C are highly similar in protein sequence, with variations mainly distributed in the C-termini. To test whether the C-terminal variations affect the P0 proteins localization in plant cells, a series of GFP tagged OsP0s were transiently expressed in rice protoplast. Examination of the fluorescence signals from the GFP-P0s showed that all of the GFP-OsP0s were localized in both the cytoplasm and nucleus (Fig. 2A), indicating that variations of the C-termini do not affect P0 protein distribution in rice cells.

### Protein–protein interactions between OsP0s and OsP1/P2 proteins

In eukaryotes, P1 directly interacted with P0, hence providing a ribosomal anchorage to P1-P2 heterodimers [20, 21]. The C-terminal fragment of P0 protein harbored the binding site for P1-P2 heterodimers [7, 9]. Homologue search of P1/P2 proteins in rice genomic database, identified a *P1* gene named *OsP1* and six *P2* genes named OsP2A-F. Gene expression data from the public RNA-seq database showed that *OsP1* and *OsP2A-C* were expressed in most of rice tissues, while the transcripts of *OsP2D-F* could not be detected in most of the tested tissues (Fig. S2B, C). *OsP2A* and *OsP2C* presented higher expression levels compared to the other *OsP2* genes (Fig. S2C), suggesting they are probably the major *OsP2* isoforms in rice. As we mentioned above, the rice P0 family proteins differ from each other mainly in the C-terminus that is important for interaction with partner proteins. Thereby, we examined the protein–protein interactions of OsP0s with OsP1, OsP2A and OsP2C by yeast two hybrid assay. The results showed that all the three OsP0s interacted with OsP1, but not OsP2A and OsP2C (Fig. 2B), indicating the interactions of P0 with the P1/P2 complex were evolutionarily conserved, and the variations of the C-termini of OsP0s did not affect the interactions.

### Gene expression patterns of *OsP0s*

**Fig. 3:**
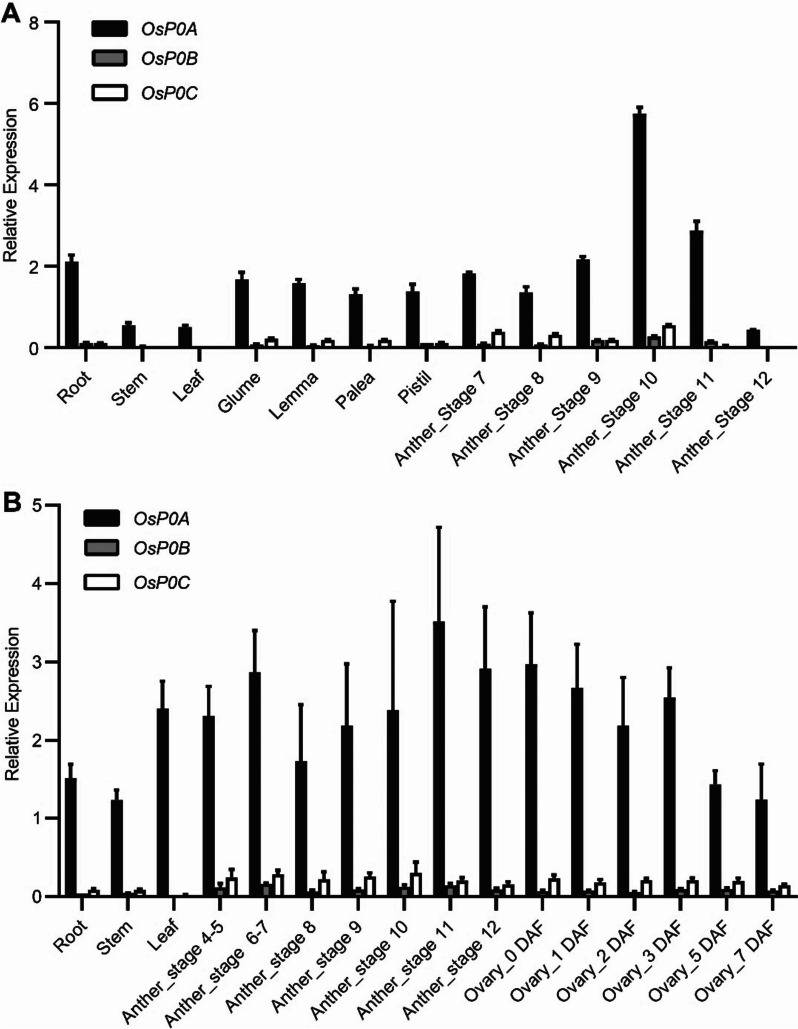
Expression of *OsP0A*, *OsP0B* and *OsP0C*. Vegetative tissues were collected from HHZ (**A**) and WYG (**B**) at heading stage. Anthers were collected from stage 4–5 to stage 12. Ovaries were collected from WYG before pollination and 1, 2, 3, 5, and 7 d after pollination. The levels of *OsP0A*, *OsP0B*, and *OsP0C* transcripts were determined by qRT-PCR using *OsUbq5* as an internal control. Data are shown as means ± SD (*n* = 3)

To understand the role of *OsP0s* in rice development, we examined the gene expression of *OsP0s* in the public rice RNA-seq database (http://expression.ic4r.org/) [22]. *OsP0A* was found to be expressed at a relative high level, while *OsP0B* and *OsP0C* were weakly expressed, or barely detectable in some rice tissues (Fig. S2A). We further analyzed the expression of *OsP0s* in various tissues of HHZ (an *indica* variety) by qRT-PCR. The transcripts of *OsP0A*, *OsP0B* and *OsP0C* were detected in most of the tested tissues. The *OsP0A* transcripts was more abundant, presenting ≥ 90% of the total *P0* transcripts (Fig. 3A). The same experiment was also conducted in WYG (a *japonica* variety), in which *OsP0s* displayed the similar expression patterns (Fig. 3B)*.* Ribosomal protein-encoding genes are always transcribed in a well-coordinated manner [23]. Gene co-expression analysis revealed that the co-expressed genes of *OsP0A* were enriched in the group of structural constituents of ribosome (Fig. S3). These results suggested *OsP0A* is the predominant *P0* gene in rice. There are three *P0* genes in Arabidopsis, and two *P0* genes in maize. It was surprising that in both Arabidopsis and maize, a predominant *P0* gene contributes to the majority of the *P0* transcripts (Figs. S4, S5), which was similar to the *P0* family genes in rice. These results suggested convergent evolution of *P0* genes among various plant species.

### Phenotypes of the null mutants of the rice *P0* genes

**Fig. 4:**
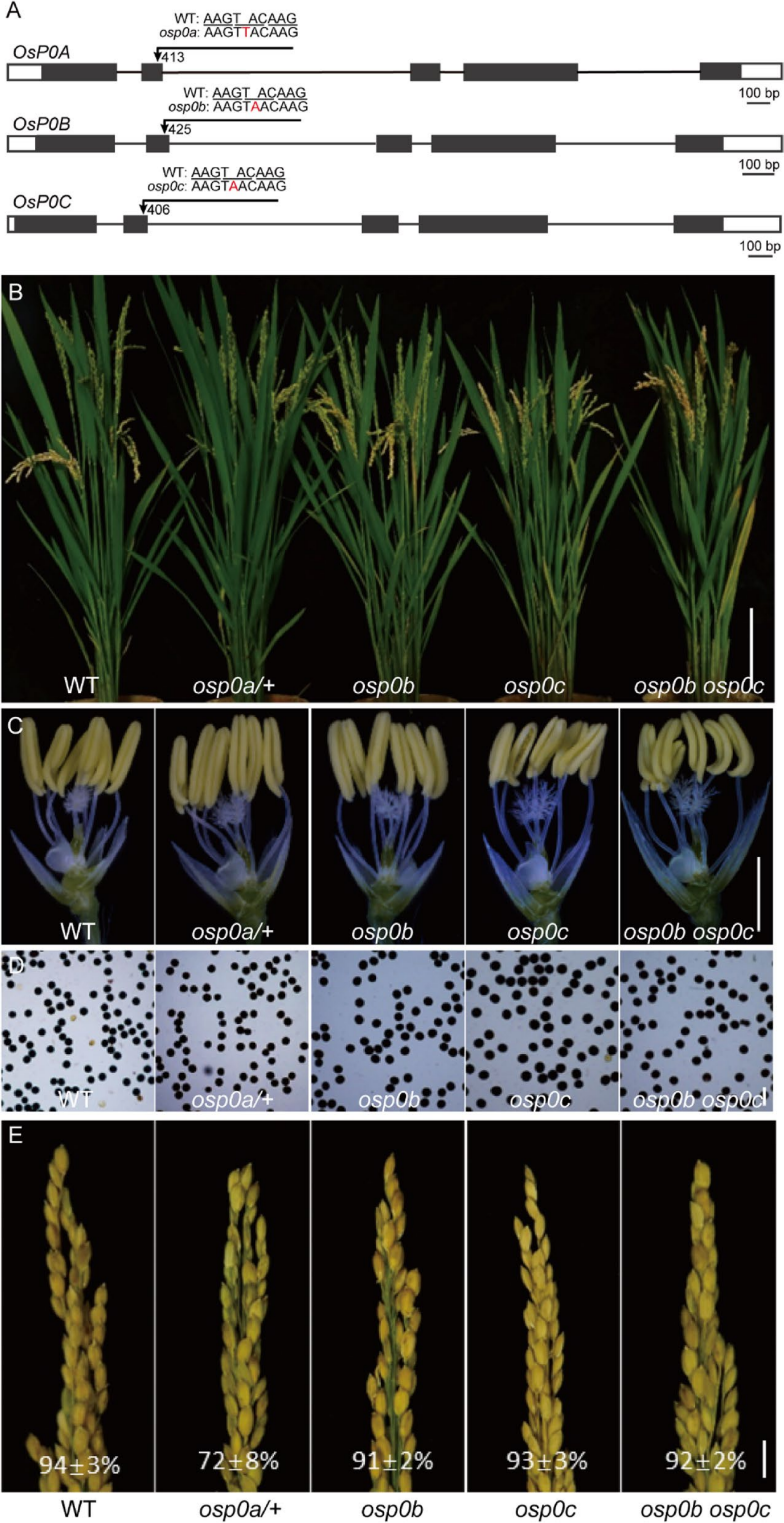
Phenotypes of *osp0a/* + , *osp0b*, *osp0c,* and *osp0b osp0c* mutants. (**A**) Structure of *OsP0A*, *OsP0B*, and *OsP0C* genes and mutation sites. (**B**-**E**) Comparison of phenotypes of WT, *osp0a/* + , *osp0b*, *osp0c,* and *osp0b osp0c* mutant plants in plant morphology (**B**), anther (**C**), pollen grains stained with I_2_–KI (**D**), and panicles with seed-setting rates (**E**). Scale bar = 10 cm (**B**), 2 mm (**C**), 100 μm (**D**) and 1 cm (**E**)

As a way to investigate the functions of *OsP0s* in plant development, we deployed the CRISPR/Cas9 technology to knock out *OsP0s* in rice. A guiding sequence was designed to target the conserved region of the three *OsP0s* in exon 2 (Fig. 4A). No homozygous mutant was obtained for *OsP0A*, but a heterozygous mutant was obtained with a single nucleotide insertion (T_413_) in exon 2 that disrupted the open reading frame (Fig. 4A). This plant (*osp0a/* +) looked normal in vegetative growth and anther development (Fig. 4B, C). The pollen grains from *osp0a/* + plants were round, and could be stained darkly with I_2_-KI solution, appearing similar to that of the WT plants (Fig. 4D). However, the seed-setting rate of *osp0a/* + plants (72 ± 8%) was significantly decreased compared with that of the WT plants (94 ± 3%) (Fig. 4E). A homozygous mutant of *osp0b* with a single nucleotide insertion in exon 2 of *OsP0B* and a homozygous mutant of *osp0c* with a single nucleotide insertion in exon 2 of *OsP0C* were identified. Both of these mutations disrupted the corresponding genes, but the homozygous mutants displayed normal vegetative growth, anther development, pollen appearance, and normal seed setting (Fig. 4E). We then crossed the two mutant genes together and allowed self-pollination of the F_1_ plant. Double mutant of *OsP0B* and *OsP0C* genes were isolated from the F_2_ progeny by High Resolution Melting (HRM) analysis of the mutation sites. The *osp0b osp0c* double mutant also showed similar phenotypes to the WT plants (Fig. 4B-E), suggesting that *OsP0B* and *OsP0C* did not play a significant role in the plant growth and development.

### Embryo development of *osp0a/* + in rice

Failure in male and female gametophyte development and embryo abortion could all lead to the absence of homozygous mutant progeny from self-pollination. To differentiate these possibilities, we first examined the seeds derived from self-pollination of the heterozygous *osp0a/* + plants. Genotyping of the progenies of *osp0a/* + plants obtained 57 WT plants, 126 heterozygous *osp0a/* + plants and 0 homozygous *osp0a* plants, suggesting the homozygous *osp0a* seeds were aborted during development. The ratio of WT plants and heterozygous *osp0a/* + plants was consistent with the expected value 1:2 of a single gene (Table 1). We further used the heterozygous *osp0a/* + plant as pollen donor to cross-pollinate the male sterile mutant *osnp1-2* that carried a homozygous mutation of the *OsNP1* gene [24]. 52 WT plants and 43 heterozygous *osp0a/* + plants were obtained from the F_1_ generation. The χ^2^ value of the F_1_ population fall well within the range of that expected for the ratio 1:1 (Table 1). We finally used the heterozygous *osp0a/* + plants as female parent to cross with WT WYG (Table 1). Again, the segregation data also fit the hypothesis that gametophyte development was normal in *osp0a/* + plants (Table 1). Together, these results suggested that *osp0a* homozygous mutation may cause embryo lethality.

**Table 1:** Progeny genotypes from self-pollination and reciprocal pollination of the *osp0a/* + plant

Pollination types	Progeny genotype (W:H:M)	χ^2^ (χ^2^_0.95_ = 3.841)
*osp0a/* + self-pollination	57:126:0 (expect, 1:2:0)	0.393
*osnp1-*2(♀)x*osp0a/* + (♂)	52:43:0 (expect, 1:1:0)	0.853
*osp0a/* + (♀)xWT(♂)	51:37:0 (expect, 1:1:0)	2.227

W, H, and M represent progenies with the wild-type, *osp0a/* + , and *osp0a* homozygous genotypes

**Fig. 5:**
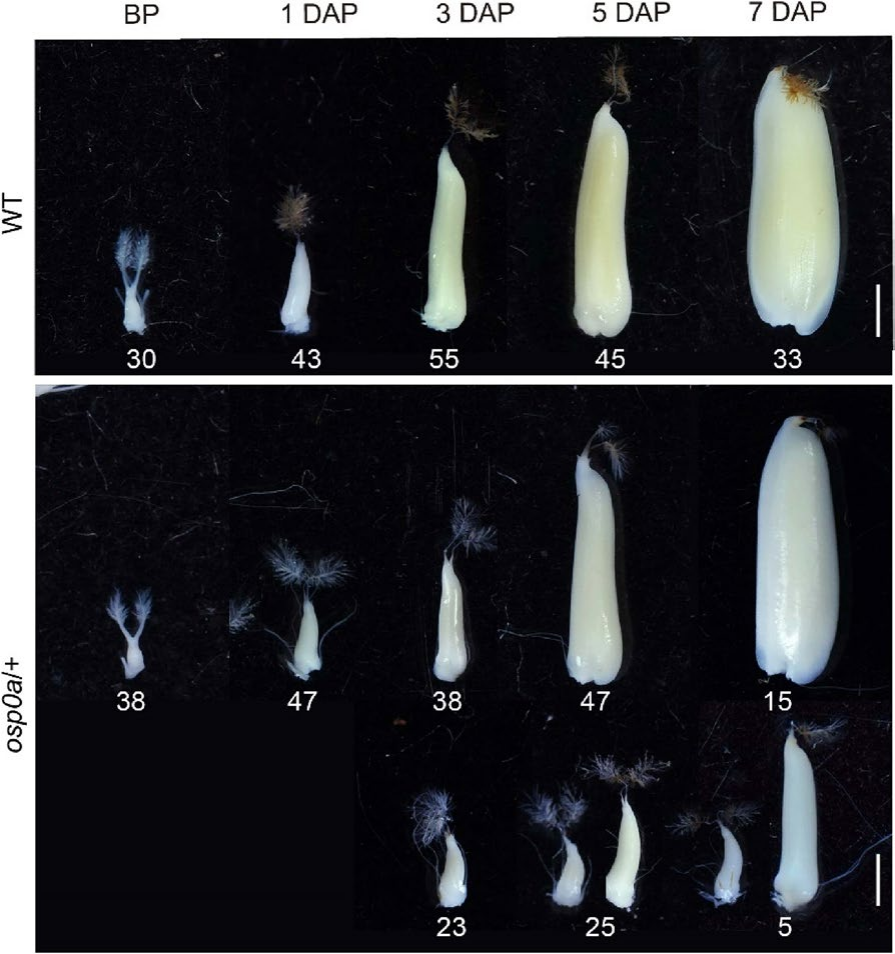
Ovule development in WT and *osp0a/* + heterozygous plant after self-pollination. WT and *osp0a/* + ovules before pollination (BP) and 1–7 day after pollination (DAP). The numbers under the ovules are numbers of samples. In *osp0a/* + , two ovules at 5 and 7 DAP were shown to represent the smallest and largest ovules in the abnormal group of samples. Scale bar = 1 mm

**Fig. 6:**
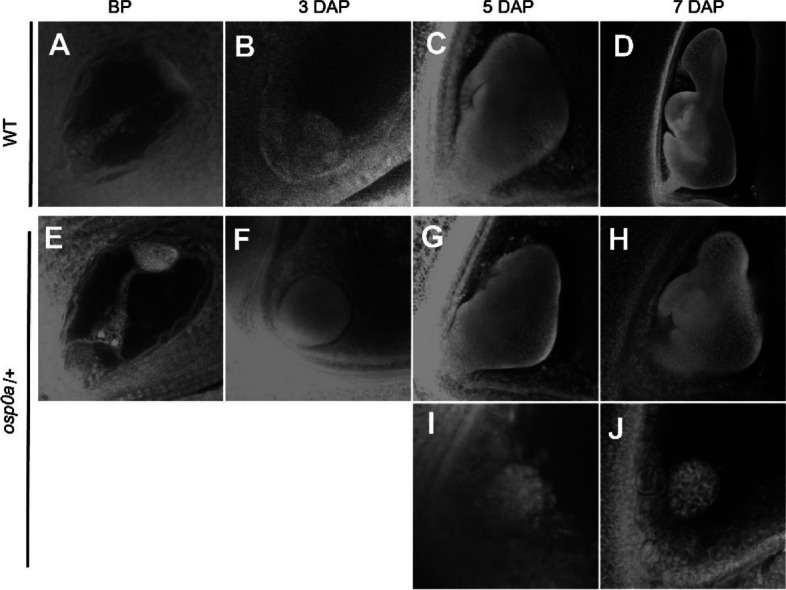
Embryo development in WT and *osp0a/* + heterozygous plant after self-pollination. Ovules were collected from the WT (**A**-**D**) and *osp0a*/ +  (**E**-**J**) before pollination (BP) and 3–7 day after pollination (DAP) and were treated with whole-mount staining to make the embryos visible. I and J show embryos inside the smallest ovules collected at 5 and 7 DAP

To determine when the abnormal embryo development occurred, we observed the ovule development following self-pollination of the heterozygous *osp0a/* + mutant. In the WT plant, the ovules gradually enlarged from 1 to 7 day after pollination (DAP) (Fig. 5). The ovules of the heterozygous *osp0a/* + plant looked similar to the WT ovules at 1 DAP, but at 3 DAP, enlargement was obviously delayed or even stopped in a portion of ovules (Fig. 5). The embryo development inside the ovule was also examined. It was clear that, embryo development stopped at the globular stage inside the abnormal small ovules but was largely normal inside the large normal ovules at 5 and 7 DAP (Fig. 6).

**Fig. 7:**
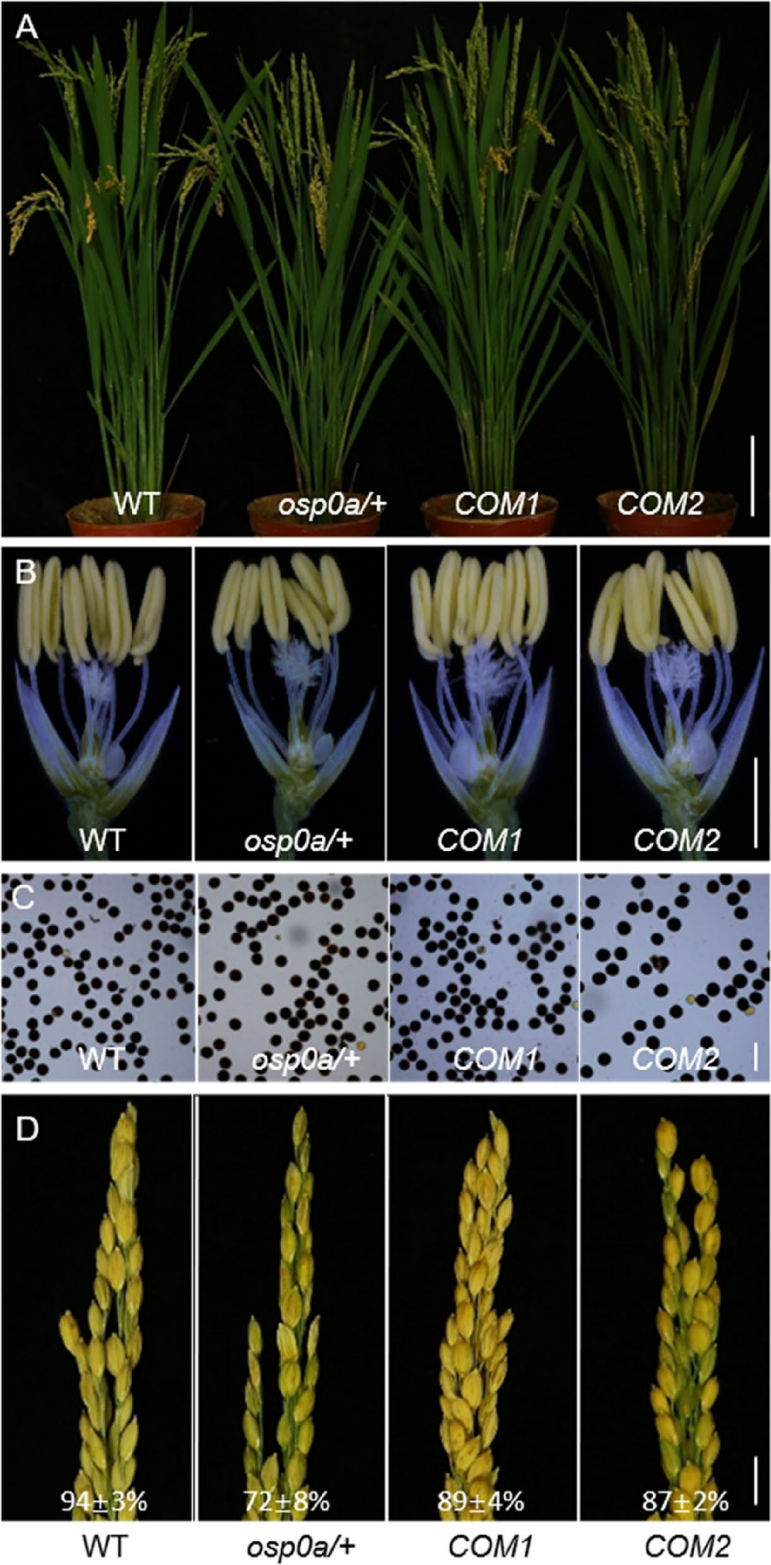
Transgenic complementation of the *osp0a* mutant. Comparison of phenotypes of WT, *osp0a/* + heterozygote, and two independent complementary lines for *osp0a* with genomic DNA. (**A**) plant morphology, (**B**) anther, (**C**) pollen grains stained with I_2_–KI, and (**D**) panicles with seed-setting rates. Scale bar = 10 cm (**A**), 2 mm (**B**), 100 μm (**C**) and 1 cm (**D**)

To further confirm that the *osp0a* seed abortion was caused by the disruption of *OsP0A*, a genomic fragment of *OsP0A* was introduced to the *osp0a/* + plants, and homozygous *osp0a* plants containing complementary genomic fragment were identified. The homozygous complementary plants (*COM1* and *COM2*) showed normal plant vegetative growth, anther development and pollen appearance (Fig. 7A-C). However, the seed-setting rates of the complementary plants (89 ± 4% and 87 ± 2%) were lower than that of the WT plants (94 ± 3%) (Fig. 7D), indicating a more appropriate genomic fragment of *OsP0A* is required for fulfilling the function of *OsP0A*. Taken together, these data suggested *OsP0A* is essential for embryo development in rice.

### Evolution of *OsP0s* in rice genomes

**Fig. 8:**
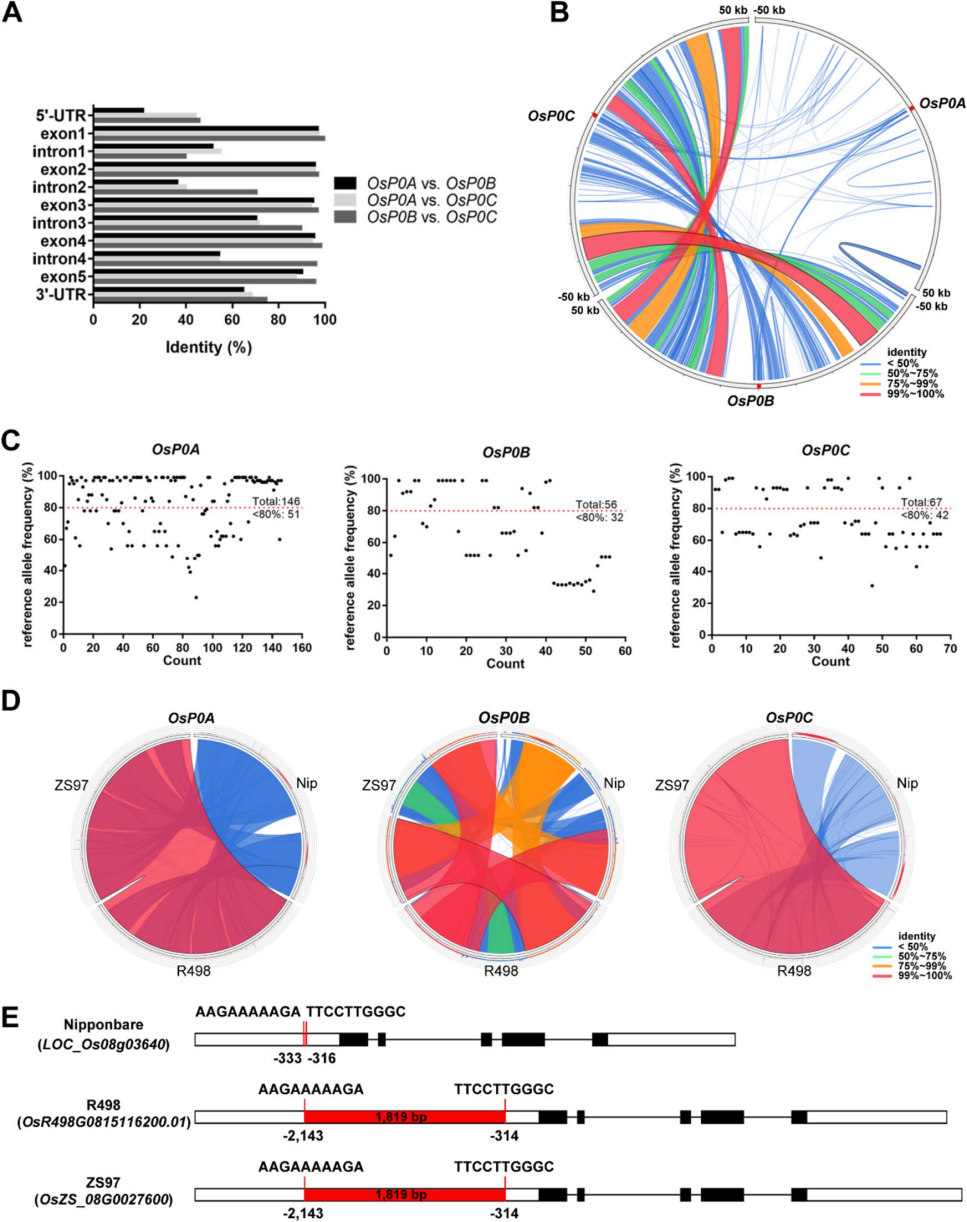
Characteristics of *OsP0A* and its family members in rice genome. (**A**) Gene structures *OsP0A*, *OsP0B* and *OsP0C* and their sequence identity. Black boxes, exon; white boxes, UTR; black lines, intron. The sequences of *OsP0A*, *OsP0B* and *OsP0C* were aligned with ClustalW for calculating identities for each exon, intron, 5’-UTR, and 3’-UTR. (**B**) Synteny analysis of the genomic regions harboring *OsP0A*, *OsP0B*, and *OsP0C*. The 50 kb genomic fragments flanking *OsP0A*, *OsP0B* and *OsP0C* were extracted from the Nipponbare genome and aligned to each other. The results were visualized with Circoletto. The position of *OsP0A*, *OsP0B* and *OsP0C* were marked with red boxes. Red lines, 99 ~ 100% identity; orange lines, 75 ~ 99% identity; green, 50 ~ 75% identity; blue lines, < 50% identity. (**C**) Variations in *OsP0A*, *OsP0B*, and *OsP0C*. The variations in the *OsP0A*, *OsP0B* and *OsP0C* genic regions and 2 kb upstream and 1 kb downstream of each gene were downloaded from the RiceVarMap2. Red lines represent the threshold of 80% reference allele frequency. (**D**) Synteny analysis of *OsP0A*, *OsP0B* and *OsP0C* in Nipponbare, R498 and ZS97 genomes. The alignments of flanking 50 kb sequences among the three rice genomes were visualized with Circoletto. Red lines, 99 ~ 100% identity; orange lines, 75 ~ 99% identity; green, 50 ~ 75% identity; blue lines, < 50% identity. (**E**) Characteristics of *OsP0A* in Nipponbare, R498 and ZS97 genomes. The red boxes represent the 1,819 bp transposon insertion in the promoters of *OsP0A* in R498 and ZS97. The sequences flanking the transposon insertion were shown for each rice genome

Genome annotation indicated that all of *OsP0A*, *OsP0B* and *OsP0C* had five exons and four introns (Fig. 8A). However, comparison of the genomic DNA sequences of *OsP0A*, *OsP0B*, and *OsP0C* indicated that *OsP0B* and *OsP0C* are more similar in sequence, and the sequence identity between *OsP0A* and *OsP0C* is higher than that between *OsP0A* and *OsP0B* (Fig. 8A). Syntenic analysis of 50 kb genomic sequences flanking the three genes indicated that *OsP0B* and *OsP0C* are located in large duplicated genomic regions that have little synteny with the genomic region harboring *OsP0A* (Fig. 8B). Sequence variation search indicated more variants in *OsP0A* than in *OsP0B* and *OsP0C* among the 4,726 rice varieties downloaded from the RiceVarMap2 database [25], especially the numbers of variations with reference allele frequency < 80% (Fig. 8C). These results suggested that *OsP0A* was probably evolved earlier before the duplication of genomic regions harboring *OsP0B* and *OsP0C*.

Other than the Nipponbare (NIP) genome, there are two assembled *indica* rice genomes (R498 and ZS97) available [26]. Alignment of the 50 kb genomic sequences flanking the three genes among the *indica* and *japonica* genomes indicated high synteny for all the three loci (Fig. 8D), indicating the presence of the three genomic fragments in *Oryza sativa* ancestry plant before differentiation of *indica* and *japonica* subspecies. Sequence alignment identified an 1819 bp mutator transposon sequence in the promoter region of *OsP0A* in R498 and ZS97 genomes but absent in NIP genome (Fig. 8E). We also PCR-amplified the *OsP0A* genomic DNA from HHZ, an *indica* variety and WYG, a *japonica* variety that were used as experimental materials in this study. Sequence analysis indicated that the *OsP0A* genomic DNA in HHZ, WYG, and R498 were identical, and all of them have the transposon sequence in the promoter. The transposon sequence was likely inserted into the *OsP0A* promoter after the splicing of *indica* and *japonica* subspecies, and crossbreeding between *indica* and *japonica* materials moved the gene across subspecies. To determine if the transposon insertion has an impact on the gene expression, the *OsP0A* transcript levels were examined in different tissues collected from the three varieties. The *OsP0A* mRNA levels were slightly higher in NIP than in HHZ and WYG (Fig. S6), suggesting that the transposon insertion does not have a big impact on the gene expression.

## Discussion

### *OsP0A* is essential for rice embryo development

Protein biosynthesis conducted by ribosome is a basic biological event in all living cells. Ribosome is a well-organized apparatus composed of rRNAs and a large number of ribosomal proteins [27]. Losses of ribosomal proteins cause phenotypes with various severity, which are largely dependent on the degree of damage on the ribosome activity [28]. One typical feature of the mutants of the conserved cytoplasmic ribosomal protein-encoding genes is embryo lethal. For instance, the mutants of each gene of *RPS5A*, *RPS11A*, *RPL3A*, *RPL8A*, *RPL10A*, *RPL19A*, *RPL23C* and *RPL40B* presented severe embryo-lethal phenotypes in Arabidopsis [29–31]. In these mutants, embryos appeared normal before globular stage, but could not advance any further [29–31]. P0 protein together with P1/P2 proteins form the stalk of ribosome in eukaryote, which is indispensable for ribosomal function in yeast and animal [3]. In the absence of P0 protein, a deficient 60S ribosome was assembled, which was impaired in protein synthesis resulting in cell death in yeast [17]. Gene silencing analysis demonstrated that the ribosomal protein P0 is required for the viability of *Haemaphysalis longicornis* [32]. In this study, three *P0* family genes named *OsP0A*, *OsP0B* and *OsP0C* were identified in rice. Among the three *P0* genes, *OsP0A* was the predominant gene expressed in all the tested tissues, whereas *OsP0B* and *OsP0C* were expressed at very low levels (Fig. S2A, Fig. 3). Consistently, knockouts of *OsP0B* or *OsP0C* or both of them had no visible impact on the plant growth and development, while knockout of *OsP0A* resulted in embryo-lethality (Figs. 4, 5). Cytological analysis showed that the *osp0a* mutant embryos were arrested at globular stage (Fig. 6), which was consistent with the typical phenotypes of the essential ribosomal protein mutants [27]. The embryo-lethal phenotype is expected because P0 protein is required for ribosome function, and *OsP0A* as the predominant *P0* gene in rice, when knocked out, the mutation would certainly impair the ribosome function, and in turn, abolish the protein synthesis.

### The evolution of *P0* family genes in rice

Gene duplication provides resources for further evolution of the family members with inherited function, specialized function or loss of function, and/or evolved new functions [28, 33]. In plants, each cytoplasmic ribosomal protein is encoded by a gene family comprised of two or more homologous members. For example, 213 and 235 cytoplasmic ribosomal protein encoding genes were identified in Arabidopsis and rice respectively, and these genes were categorized into 81 gene families in both species [1]. This enormous increase in members of each cytoplasmic ribosomal protein family makes the ribosomes highly heterogeneous, and provides freedom for gene evolution [33]. For instance, RPL10 is an essential integral component of the large subunit of eukaryotic ribosomes. There are three *RPL10* paralogs in Arabidopsis, *AtRPL10A*, *AtRPL10B* and *AtRPL10C*. All of them could complement a yeast mutant in *RPL10*, indicating that they are functional in translation [34]. However, *AtRPL10A* and *AtRPL10B* appearing to be more important in plant development and *AtRPL10A* appeared to play a major role in the regulation of protein synthesis under UV-B stress [30]. Arabidopsis has three *RPL9* genes, *AtRPL9B*, *AtRPL9C*, and *AtRPL9D*. Although these genes are ubiquitously expressed, the transcript of *AtRPL9C* is more abundant, and is twofold and threefold higher than that of *AtRPL9D* and *AtRPL9B* respectively [35]. The knockdown mutant of *atrpl9c/pgy* displayed pointed leaves and more prominent marginal serrations [35], while the null mutant *atrpl9d* showed no visible differences from the wild type [36]. However, the double mutant *atrpl9c atrpl9d* was embryo lethal [36], suggesting a dosage-dependent requirement of *RPL9* for ribosome activity. The ribosomal P0 proteins are very conserved in eukaryotic kingdom. Heteroexpression of *P0* genes from worm, mammals, or protozoa could complement the genetic defect of *P0* gene in yeast [13–15]. In the present study, we identified three *P0* genes in rice. Syntenic analysis revealed that the presence of the three *P0* genes in rice ancestor before differentiation of *indica* and *japonica* subspecies, and *OsP0B* and *OsP0C* are located in large duplicated genomic regions (Fig. 8B). Moreover, *OsP0A* presented more sequence variants than *OsP0B* and *OsP0C* in a larger collection of rice varieties (Fig. 8C), indicating the duplication event of *OsP0B* and *OsP0C* happened after the emergence of *OsP0A*. Additionally, *OsP0A* was more similar to *OsP0C* than to *OsP0B in* DNA sequences*.* These data suggest *P0* gene has undergone two gene duplication events in rice. *OsP0A* may be the parent copy, and the first duplication event gave rise to *OsP0C*, and *OsP0B* emerged from a duplication of *OsP0C*. A transposon was found in the promoter of OsP0A in *indica* varieties, but not in the *japonica* variety NIP (Fig. 8D), indicating the transposon was likely inserted into the OsP0A promoter after the splicing of *indica* and *japonica* subspecies. The presence of the transposon in the promoter of OsP0A in WYG might come from the crossbreeding between *indica* and *japonica* materials.

The OsP0A, OsP0B, and OsP0C share high identities in amino acid sequences. Consistent with the relationship of P0 and P1 in other eukaryotes, OsP0s directly interacts with OsP1 (Fig. 2B). The yeast P0 protein was reported to be localized in cytoplasm and proposed to be incorporated into the 60 s ribosomal subunit in the late cytoplasmic stage of ribosome assembly [19]. However, all the OsP0s appeared to be located in both cytoplasm and nucleus (Fig. 2A), which was the same as the HsP0 distribution in human cells [37], providing the possibilities that OsP0s and HsP0 may be assembled onto pre-ribosomes in nucleus. The transcripts of *OsP0A* presented ≥ 90% of the total *P0* transcripts in most of the tested rice tissues (Fig. 3). The null mutant of *OsP0A* was embryo lethal (Fig. 6), while knockout of *OsP0B* and *OsP0C* simultaneously did not show any visible phenotype in rice (Fig. 4). These results suggested that *OsP0A* is the predominant P0 gene in rice.

In addition to the fundamental functions in protein translation, lots of ribosomal proteins evolved extraribosomal functions in plants, such as RPL10A, RPL24B, RPS6, and the organelle ribosomal protein uL18-L1, uL18-L8, OsPRPL18 and so on [38–42]. The eukaryotic P0 family were also documented to be functional in ribosome-independent manners. The virus protein VPg (viral protein genome-linked) protects the RNA of *Potato virus A* from degradation and facilitats its translation [43]. The tobacco NbP0 was essential for the activity of VPg and, together with VPg and eIF(iso)4E synergistically enhanced viral translation [44]. Biochemical analysis confirmed that the *Drosophila* ribosomal protein P0 contained apurinic/apyrimidinic endonuclease activity for both single- and double-stranded DNA, and might act as a DNA repair protein [45]. NONO is an essential component of paraspeckle, playing a pivotal role in the repair of DNA double-strand breaks (DSB) [46]. The human HsP0 directly bind to the RRM1 and RRM2 domains of NONO, therefore enhancing non-homologous end joining-mediated DSB repair [47]. OsSec18 is a conserved ATPase required for vesicle membrane fusion [48]. OsSec18 interacts with Os60sP0/OsP0A and together with other proteins constitutes a 290-kDa complex in rice endosperm cells. However, the function of OsP0A in the complex remains unclear [48]. As P0 proteins are highly conserved, and OsP0s localizes both in cytoplasm and nucleus, we speculate that OsP0s probably also have evolved extraribosomal functions as its homologs did. However, more studies are required to support this notion.

In summary, we here demonstrate the essential role of *P0* family genes in plant survival and provide the evidences that *OsP0A* is the predominant *P0* gene in rice. Also, an evolutionary model is proposed that *OsP0C* is duplicated from *OsP0A*, and *OsP0B* is duplicated from *OsP0C*. Together, our data highlight the functions of P0 family genes in plants.

## Materials and methods

### Plant materials and growth conditions

The *japonica* variety Wuyungeng 7 (WYG) was kindly provided by Dr. Jianmin Wan (Nanjing Agricultural University). The *indica* variety Huanghuazhan (HHZ) was obtained form the Rice Research Institute of Guangdong Academy of Agricultural Sciences. WYG and HHZ were used as the wild-type in this study. Rice seeds were sterilized by 0.3% hydrogen peroxide, and then germinated in pans. The seedlings at the 4–5 leaf stage were transplanted into the paddy field with regular care.

### Phylogenetic analysis

P0 family proteins from *Cyanidioschyzon merolae*, *reinhardtii*, *Chara braunii*, *Selaginella moellendorffii*, *Physcomitrium patens*, *Solanum lycopersicum*, *Arabidopsis thaliana*, *Medicago truncatula*, *Zea mays* and *oryza sativa* were obtained from the Ensembl Plants Database (http://plants.ensembl.org/index.html) with the yeast P0 protein sequence as a query. Protein multiple sequence alignment was performed using ClustalW, and the neighbor-joining tree was constructed using MEGA11 with default parameters [49].

### Protein subcellular localization analysis

Plasmids comprising the expression cassettes *GFP-OsP0A*, *GFP-OsP0B*, and *GFP-OsP0C* driven by *35S* promoter were constructed respectively. The resultant plasmids together with the nuclear marker *ARF19IV-mCherry* were introduced into rice protoplast by the polyethylene glycol (PEG)–calcium mediated method as previous described [50]. After 12 h incubation, the fluorescence signal in the transformed protoplasts were examined by a laser confocal scanning microscope (LSM-800; Carl Zeiss).

### Yeast two hybrid assay

The CDSs of *OsP0A, OsP0B,* and *OsP0C* were individually cloned into *pGBKT7* (containing a GAL4 DNA-binding domain, BD construct), and the CDSs of *OsP1 OsP2A* and *OsP2C* were cloned into *pGADT7* (containing a GAL4 activating domain, AD construct) respectively. Each of the resultant BD constructs in combination with the various AD constructs were introduced into the yeast strain *AH109*. The transformed yeast cells were further plated on the -LT (SD-Leu/-Trp) medium. The positive clones were transferred on the -LTHA (SD-Leu/-Trp/-His/-Ade) medium for testing the protein–protein interactions.

### RNA extraction and qRT-PCR assay

For gene expression analyses, qRT-PCR assay was performed as previously described [51]. Briefly, total RNA was extracted from various rice tissues and used for cDNA synthesis. qPCR was performed using SYBR Premix Ex Taq II (TaKaRa, Dalian, China) with gene specific primers (Table S1). The *OsUbq5* gene was employed as a normalizing reference gene.

### Generation of *p0* mutants and complementation of* p0a* mutant

The plasmid and method described by Liu et al. [52] were deployed for construction of the CRISPR/Cas9 gene knockout plasmid to create mutations in *OsP0A*, *OsP0B,* and *OsP0C* genes with the target site sequence AGTACAAGGTATAACTGGCA.

To generate the Com-*OsP0A* vector for *p0a* mutant complementation, the *OsP0A* genomic fragment, including 4 kb upstream promoter and 1.4 kb downstream region was PCR-amplified using Com-OsP0A-F/R primers. The PCR product was cloned into binary vector *pCAMBIA1300* using In-Fusion HD Cloning Kit (Takara, Dalian, China).

All the constructs were sequence-confirmed before introduced into the *Agrobacterium tumefaciens* AG10 strain for rice transformation. The transgenic plants were examined for mutations in *OsP0A*, *OsP0B,* and *OsP0C* genes by High Resolution Melt (HRM) method [53] with primer sets osp0a-HRM-F/R, osp0b-HRM-F/R, and osp0c-HRM-F/R, respectively. To identify the background genotype of *osp0a* complementary transgenic plants, primer sets Com-OsP0A-1-BJ-F/R and Com-OsP0A-2-BJ-F/R were used to amply the specific genomic fragment covering the mutation site in *osp0a/* + mutant*.* The PCR product was diluted 1000–2000 times for HRM assay using the primers osp0a-HRM-F/R. All the primers were listed in Table S1.

### Synteny analysis

The sequences for *OsP0A*, *OsP0B*, and *OsP0C* gene were retrieved from the Nipponbare genome (Release 7 of the MSU Rice Genome Annotation Project, http://rice.plantbiology.msu.edu/) and aligned with ClustalW for calculating identities for each exon, intron, 5’-UTR, and 3’-UTR. The promoter sequences upstream of *OsP0A* were extracted from the Nipponbare, ZS97 and R498 genomes [54, 55] for characterization of the transposon insertion. The 50 kb genomic fragments flanking *OsP0A*, *OsP0B* and *OsP0C* were extracted from the Nipponbare, ZS97 and R498 genomes. Alignments of the flanking 50 kb sequences were performed and visualized with Circoletto [56]. The variations in the *OsP0A*, *OsP0B* and *OsP0C* genic regions and 2 kb upstream and 1 kb downstream of each gene were downloaded from the RiceVarMap2 (http://ricevarmap.ncpgr.cn/) [25].

### Supplementary Information

**Additional file 1:** **Fig. S1.** Multiple sequence alignment of plant P0 proteins. **Fig. S2.** The gene expression patterns of *OsP0s*, *OsP1*, and *OsP2s* in rice. The expression data were retrieved from the public RNA-seq database (http://expression.ic4r.org/). **Fig. S3.** GO enrichment of the co-expression genes of *OsP0A*. The co-expression genes of OsP0A were predicted by riceFREND (http://ricefrend.dna.affrc.go.jp/), and the top 100 ranking co-expression genes were selected for GO analysis. **Fig. S4.** The gene expression patterns of *AtP0s* in Arabidopsis. The expression data were retrieved from the public gene expression database (https://bar.utoronto.ca/efp_arabidopsis/cgi-bin/efpWeb.cgi).**Fig. S5.** The gene expression patterns of *ZmP0s* in maize. The expression data were retrieved from the public gene expression database (https://maizemine.rnet.missouri.edu/maizemine/begin.do).**Fig S6.** Comparison of the *OsP0A* gene expression in different rice varieties. Root, shoot and leaf tissues were collected from the 5-week-old plants of WYG, NIP and HHZ. The levels of *OsP0A* transcripts were determined by qRT-PCR using *OsUbq5* as internal control. Data are shown as means ± *SD* (*n*=3). **Table S1.** Primers used in this study.

## Data Availability

The datasets supporting the conclusions of this article are included within the article and its additional files.
