# Supplementary material for: The ribosomal protein P0A is required for embryo development in rice
Source: BMC Plant Biol. 2023 Oct 5;23:465. doi: 10.1186/s12870-023-04445-y (PMC10552409; doi:10.1186/s12870-023-04445-y)
Supplement: Supplementary file 1 — Additional file 1: Fig. S1. Multiple sequence alignment of plant P0 proteins. Fig. S2. The gene expression patterns of OsP0s, OsP1, and OsP2s in rice. The expression data were retrieved from the public RNA-seq database (http://expression.ic4r.org/). Fig. S3. GO enrichment of the co-expression genes of OsP0A. The co-expression genes of OsP0A were predicted by riceFREND (http://ricefrend.dna.affrc.go.jp/), and the top 100 ranking co-expression genes were selected for GO analysis. Fig. S4. The gene expression patterns of AtP0s in Arabidopsis. The expression data were retrieved from the public gene expression database (https://bar.utoronto.ca/efp_arabidopsis/cgi-bin/efpWeb.cgi).Fig. S5. The gene expression patterns of ZmP0s in maize. The expression data were retrieved from the public gene expression database (https://maizemine.rnet.missouri.edu/maizemine/begin.do).Fig S6. Comparison of the OsP0A gene expression in different rice varieties. Root, shoot and leaf tissues were collected from the 5-week-old plants of WYG, NIP and HHZ. The levels of OsP0A transcripts were determined by qRT-PCR using OsUbq5 as internal control. Data are shown as means ± SD (n=3). Table S1. Primers used in this study. [file 12870_2023_4445_MOESM1_ESM.docx]

**Supplementary data**


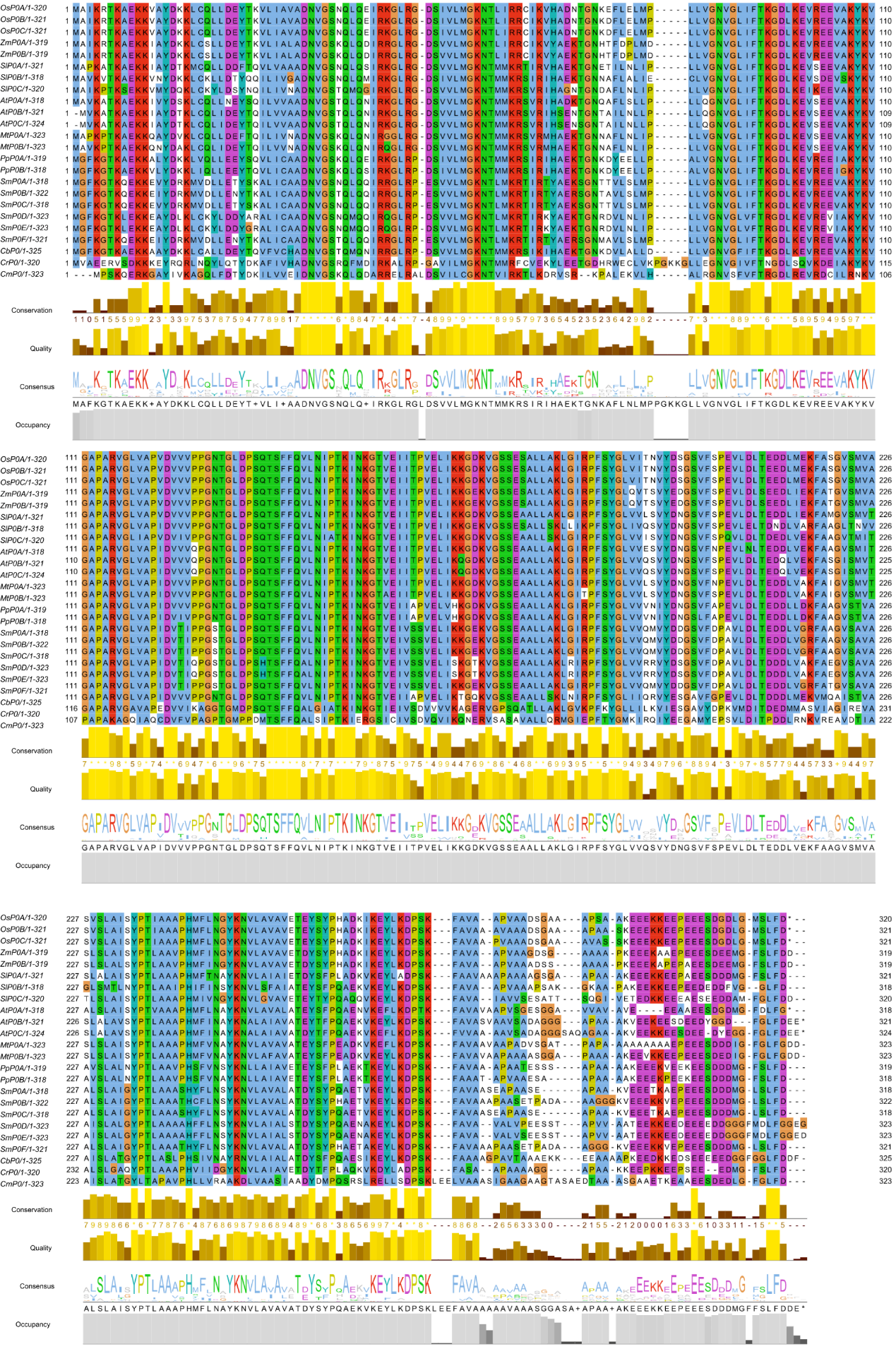


**Fig. S1. Multiple sequence alignment of plant P0 proteins.**


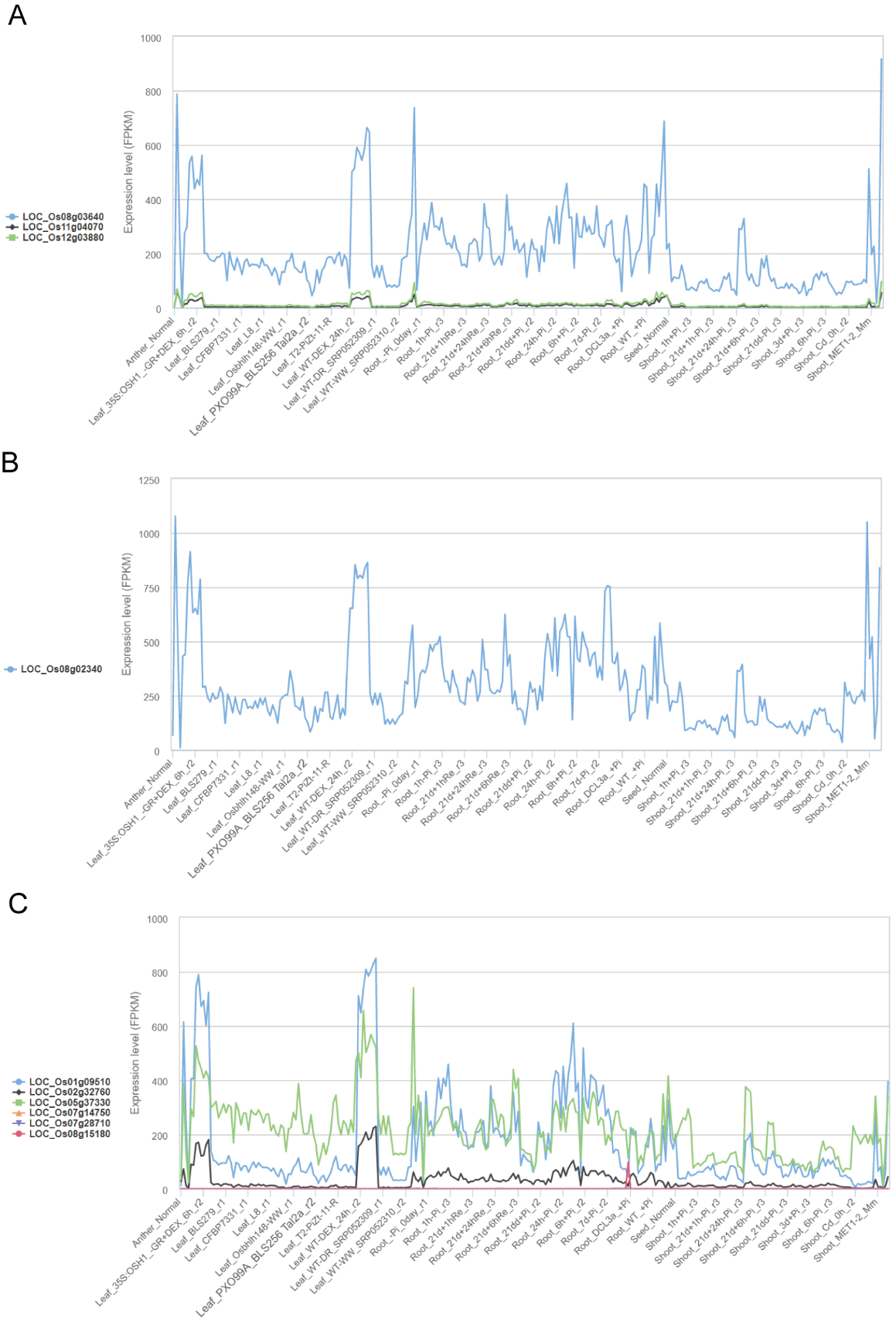


**Fig. S2. The gene expression patterns of *OsP0s*, *OsP1*, and *OsP2s* in rice.** The expression data were retrieved from the public RNA-seq database (http://expression.ic4r.org/).


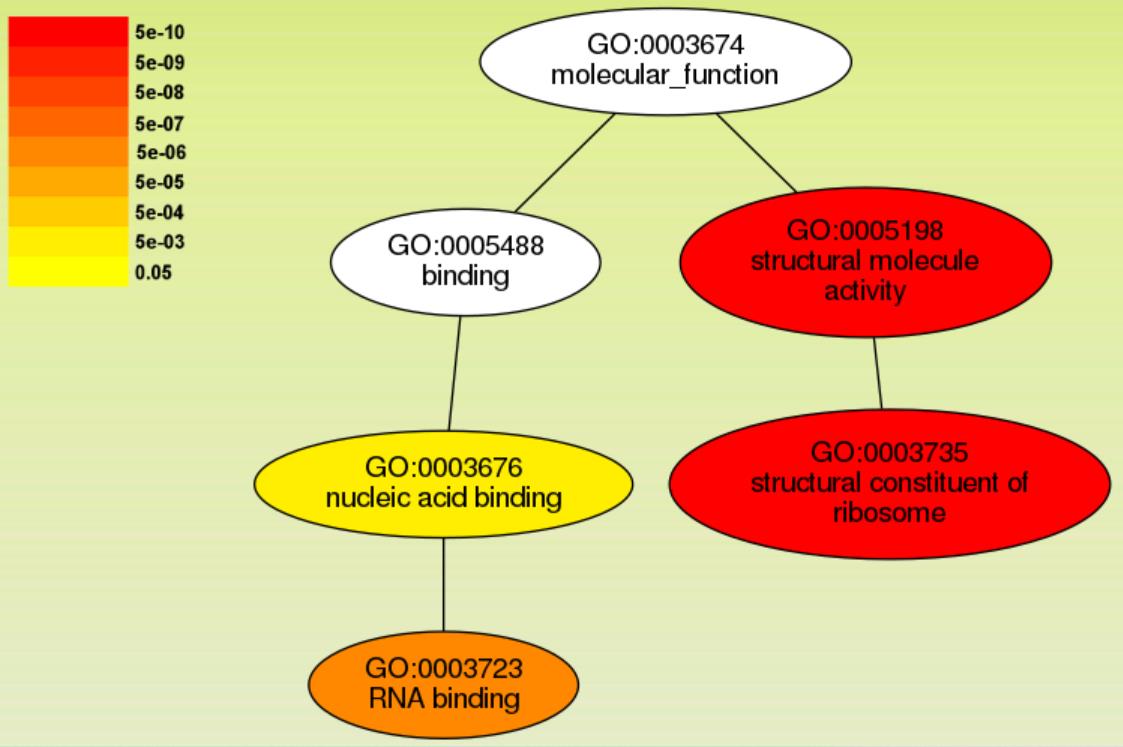


**Fig. S3. GO enrichment of the co-expression genes of *OsP0A*.** The co-expression genes of OsP0A were predicted by riceFREND (http://ricefrend.dna.affrc.go.jp/), and the top 100 ranking co-expression genes were selected for GO analysis.


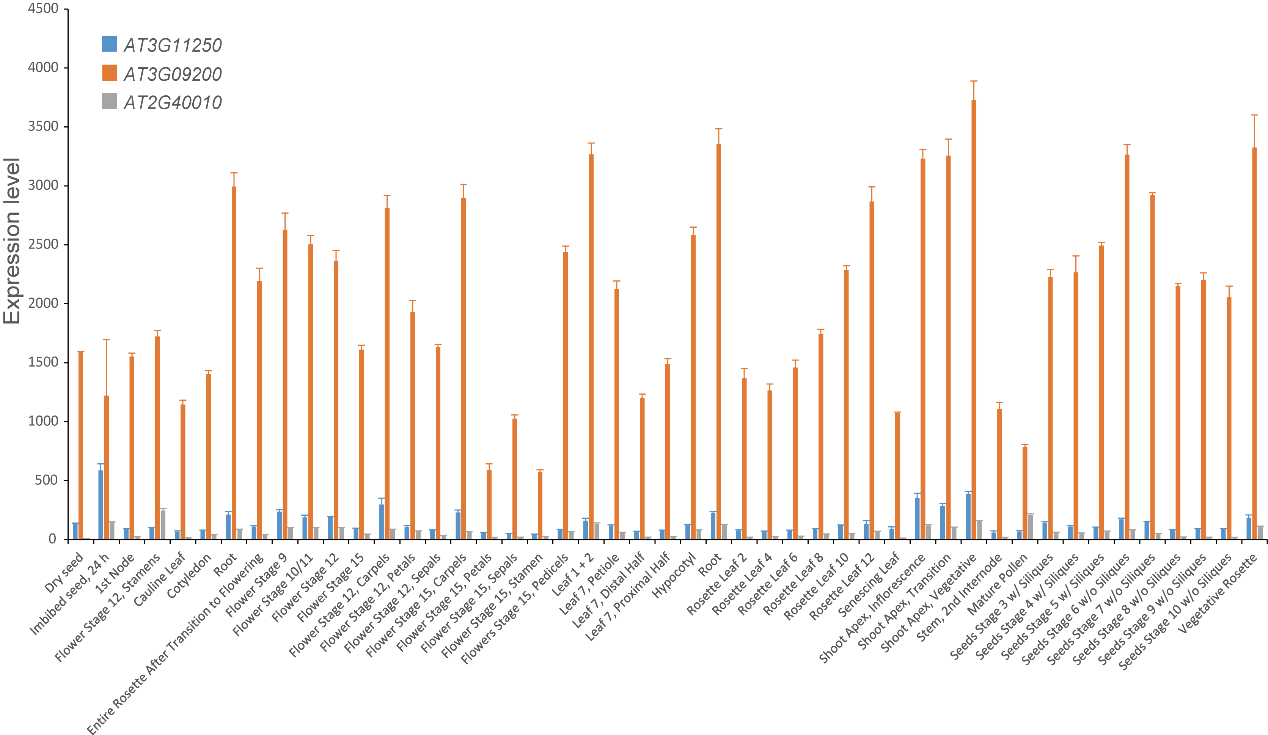


**Fig. S4. The gene expression patterns of *AtP0s* in Arabidopsis.** The expression data were retrieved from the public gene expression database (https://bar.utoronto.ca/efp_arabidopsis/cgi-bin/efpWeb.cgi).


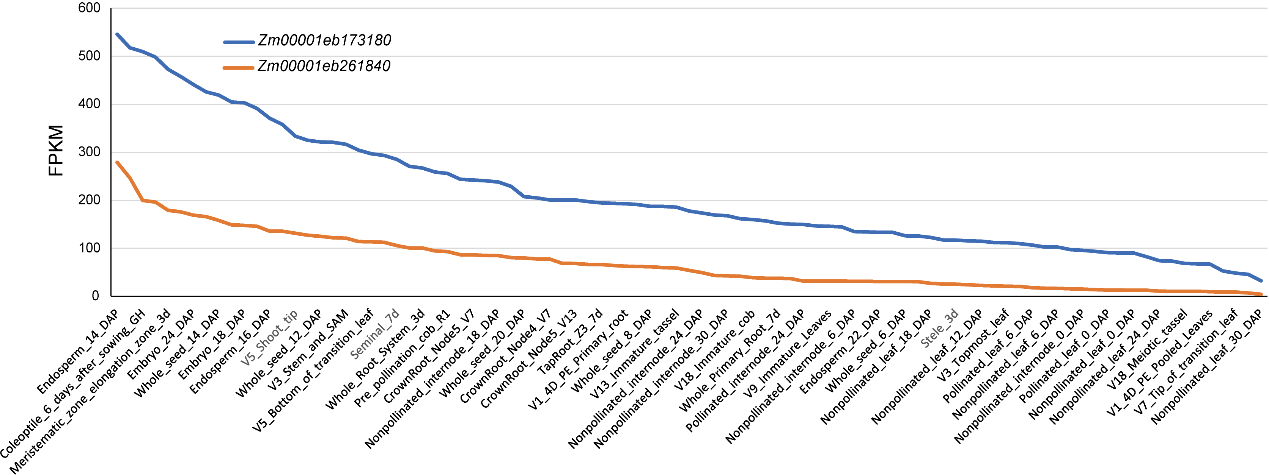
 **Fig. S5. The gene expression patterns of *ZmP0s* in maize.** The expression data were retrieved from the public gene expression database (https://maizemine.rnet.missouri.edu/maizemine/begin.do).


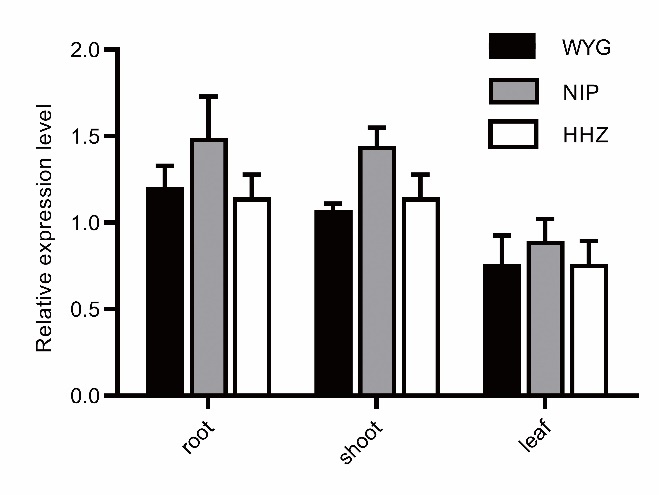


**Figure S6. Comparison of the *OsP0A* gene expression in different rice varieties**

Root, shoot and leaf tissues were collected from the 5-week-old plants of WYG, NIP and HHZ. The levels of *OsP0A* transcripts were determined by qRT-PCR using *OsUbq5* as internal control. Data are shown as means ± *SD* (*n*=3).

T**able S1. Primers used in this study**

| Primers | Sequences (5’--3’) |
| --- | --- |
| osp0a-HRM-F | ACCAAGGGTGACCTCAAGGA |
| osp0a-HRM-R | TCCATGCAGTCTAGAACACAGA |
| osp0b-HRM-F | ACCAAGGGTGACCTCAAGGA |
| osp0b-HRM-R | CTTGCAGTGTATTGAACACAG |
| osp0c-HRM-F | ACCAAGGGTGACCTCAAGGA |
| osp0c-HRM-R | GATCGATAGCCTTGTACTTGG |
| Com-OsP0A-1-BJ-F | CTTTTCCAATGCGATTTGTCTCT |
| Com-OsP0A-1-BJ-R | GATGATCCCTCCGATGTAAGTGA |
| Com-OsP0A-F | CCATGATTACGAATTCGCCCGGCCAATAGATGACGGAC |
| Com-OsP0A-R | GGCCAGTGCCAAGCTTGCTTGGCTGATGCTGCAGAGATTCC |
| Com-OsP0A-id-F | CCAGGCTTTACACTTTATGC |
| Com-OsP0A-id-R | gctattggcaattagcatgac |
| Com-OsP0A-2-BJ-F | gaaatcgatgtgatgttcttgctg |
| Com-OsP0A-2-BJ-R | ctcatataaatatacccagcttgttcac |
| UBQ-qPCR-F | CAACCAGCTGAGGCCCAAGAA |
| UBQ-qPCR-F | CCAGGGAGATAACAACGGAAGC |
| OsP0A-qPCR-F | CCTGCTCGTGTTGGTCTTG |
| OsP0A-qPCR-R | GGAGGAGCCCACCTTGTCA |
| OsP0B-qPCR-F | GACTCCATCGTCCTCATGGGC |
| OsP0B-qPCR-R | AACCTTGTACTTGGCGACCTCT |
| OsP0C-qPCR-F | ATACTCATACCCACATGCTGAC |
| OsP0C-qPCR-R | CCCAGGTCACCGTCAGATTCT |
| GFP-P0A-F | ATGGACGAGCTGTACAGATCTATGGCGATCAAGAGGACCAAGG |
| GFP-P0B/C-F | ATGGACGAGCTGTACAGATCTATGGCGATCAAGCGGACCAAGG |
| GFP-P0-R | TTGCCAAATGTTTGAACTGCAGTTAGTCGAAGAGGCTCATGCCCAGGTC |
| BD-P0A-F | AGAGGAGGACCTGCATATGATGGCGATCAAGAGGACC |
| BD-P0-R | GGATCCCCGGGAATTCTTAGTCGAAGAGGCTCATGC |
| BD-P0-B/C-F | AGAGGAGGACCTGCATATGATGGCGATCAAGCGGACC |
| AD-P1-F | ACCAGATTACGCTCATATGATGTCGTCCAGCGAGGTC |
| AD-P1-R | CACCCGGGTGGAATTCTTAGTCGAACAAGCTGAAACCCATG |
| AD-P2C-F | ACCAGATTACGCTCATATGATGAAGCTGATCGCTGCCTAC |
| AD-P2C-R | CACCCGGGTGGAATTCTTAGTCAAACAAGCTGAAACCCATG |
| AD-P2A-F2 | ACCAGATTACGCTCATATGATGAAGTTCATTTCTGCCTATCTG |
| AD-P2A-R | CACCCGGGTGGAATTCTCAGTCAAACAAACTGAAGCCCATGTC |
